# Designing Mechanical Properties of 3D Printed Cookies through Computer Aided Engineering

**DOI:** 10.3390/foods9121804

**Published:** 2020-12-04

**Authors:** Agnese Piovesan, Valérie Vancauwenberghe, Wondwosen Aregawi, Mulugeta A. Delele, Evi Bongaers, Mathijs de Schipper, Kjeld van Bommel, Martijn Noort, Pieter Verboven, Bart Nicolai

**Affiliations:** 1 Division BIOSYST-MeBioS, KU Leuven – University of Leuven, 3001 Leuven, Belgium; agnese.piovesan@kuleuven.be (A.P.); V.Vancauwenberghe@bruker.com (V.V.); waregawi@umn.edu (W.A.); muluadmasdel@gmail.com (M.A.D.); bart.nicolai@kuleuven.be (B.N.); 2 MicroCT department, Bruker, 2550 Kontich, Belgium; bongaers@bruker.com; 3 Bahir Dar Institute of Technology, Bahir Dar University, Bahir Dar 6000, Ethiopia; 4 Department Equipment for Additive Manufacturing, TNO, NL-5656 Eindhoven, The Netherlands; mathijs.deschipper@tno.nl (M.d.S.); kjeld.vanbommel@tno.nl (K.v.B.); 5 Wageningen Food & Biobased Research, Wageningen University and Research, 6708WG Wageningen, The Netherlands; martijn.noort@wur.nl

**Keywords:** additive manufacturing, texture, 3D food printing, structure, X-ray computed tomography, finite element modelling, computed aided engineering

## Abstract

Additive manufacturing or 3D printing can be applied in the food sector to create food products with personalized properties such as shape, texture, and composition. In this article, we introduce a computer aided engineering (CAE) methodology to design 3D printed food products with tunable mechanical properties. The focus was on the Young modulus as a proxy of texture. Finite element modelling was used to establish the relationship between the Young modulus of 3D printed cookies with a honeycomb structure and their structure parameters. Wall thickness, cell size, and overall porosity were found to influence the Young modulus of the cookies and were, therefore, identified as tunable design parameters. Next, in experimental tests, it was observed that geometry deformations arose during and after 3D printing, affecting cookie structure and texture. The 3D printed cookie porosity was found to be lower than the designed one, strongly influencing the Young modulus. After identifying the changes in porosity through X-ray micro-computed tomography, a good match was observed between computational and experimental Young’s modulus values. These results showed that changes in the geometry have to be quantified and considered to obtain a reliable prediction of the Young modulus of the 3D printed cookies.

## 1. Introduction

Food texture refers to the sensory experience generated by the structural, mechanical, and surface properties of foods [1,2,3]. Consumers’ liking of a food product is greatly influenced by texture perception, which needs to be properly understood [1]. Food texture is determined by both the product’s composition [4] and structure [5], which can be controlled [6]. Porosity is a structural feature that affects texture and is as such a possible design parameter to control food product texture [7,8,9]. As texture is mainly perceived through deformation of the food by manipulation or chewing, mechanical properties are often used as a proxy of texture. The Young modulus is such a property that is defined as the slope of the curve that describes the relationship between stress and the corresponding strain during deformation of the food, and it is a measure of its stiffness [3].

Additive manufacturing (AM), also referred to as 3D printing, can be applied to create food products with an arbitrary structure and, therefore, texture, while taking into account consumers’ individual nutritional requirements [10,11,12,13]. The interest in AM in many fields, including food manufacturing, is rapidly increasing thanks to the offered advantages such as the possibility of customizing objects with little material loss and ease of modifying and exchanging digital manufacturing data. AM is further associated with the possibility of using alternative food sources [10]. In AM, 3D objects are produced through subsequent deposition of layers of powder, liquid, or sheets of material, where a layer corresponds to the object’s cross-section at a given vertical coordinate [14,15].

Even though AM was exploited to print objects with complex geometries from different food materials such as chocolate, cookie dough, or mashed meat [15,16], use of the technology to design food products with desired shape and texture is still a challenge. AM techniques such as selective laser sintering [17,18] and binder jetting [19,20] can be used to produce complex 3D structures starting from powdered materials, such as sugar, chocolate, or starch, but the final products have low nutritional value, and the usable raw materials are limited [10]. Extrusion-based printing (EBP) [21,22], on the other hand, is suitable for more materials but the achievable designs have limited complexity [10]. Moreover, EBP requires post-processing steps, such as baking or cooking, when soft materials such as meat puree or cookie dough are used. These post-processing steps lead to deformation of the 3D printed geometry, thus further complicating structure design [21].

Food structure can be nondestructively identified and characterized with micrometer-resolution in 3D by means of X-ray micro-computed tomography (µCT). In X-ray µCT, an object is placed between an X-ray source and a detector that measures the X-ray attenuation arising from traveling through the object in the form of radiographs. Reconstruction algorithms [23,24] are then used to extract volume information from radiographs acquired at different angles [5,25]. Once the object geometry is identified, effective mechanical properties such as the Young modulus of the 3D printed food structure can be investigated through computational modelling. For this purpose, analytical models and finite element modelling (FEM) can be used. Analytical models consist of relatively simple relationships relating regular structure geometries to their effective properties [9,26,27]. These models can, however, result in laborious equations when nonregular structures are used and are limited to defined porosity ranges [27]. FEM, on the contrary, can be used to study a wider range of realistic structures [28,29] while considering different material constitutive models [8,30] but at the expense of requiring more computational power [31].

This study explores the use of Computer Aided Engineering (CAE) to guide the design of cookies with desired properties produced through EBP. In particular, the aim of this work was to exploit the correlation between the food structure and its Young modulus [4,5] to design cookies with desired stiffness, namely the structure resistance to deformation. Cookies with different honeycomb structures were 3D printed and baked. To account for deformation, the final printed and baked cookies were imaged through X-ray µCT and porosity was calculated. After imaging, the 3D printed cookies were subjected to compression tests to determine the resulting structure stiffness which was compared to that predicted by the FE model.

## 2. Materials and Methods

**Figure 1:**
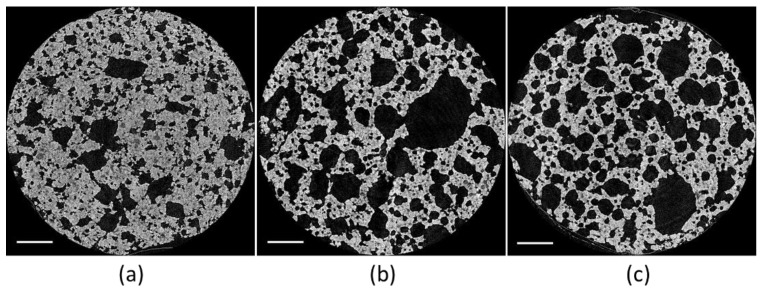
Cross-sections of cookie samples produced with recipes R1 (**a**), R2 (**b**), and R3 (**c**) (Table 1). The scale bars correspond to 800 µm. Increasing porosity can be noticed with higher baking powder concentration.

To compute the stiffness, or the effective Young modulus, of a complex structure, mechanical properties of the bulk material must be known. To this purpose, full cubic cookie material samples with sides equal to 20 mm were prepared from the cookie dough with different concentrations of baking powder (0%; 0.57%; 1.14%). The dough was formed by sheeting and cutting it into dices which were subsequently baked (170 °C, 15 min). The resulting cubic cookies were subjected to compression tests (Table 1). X-ray µCT was used to visualize the microstructure of the cookie material samples (Figure 1) and to compute the porosity (Table 1). The samples were scanned with 4.87 µm resolution in a Skyscan 1172 µCT scanner (Bruker microCT, Kontich, Flanders, Belgium); 450 projections were acquired with a voltage of 59 kV and a current of 167 µA. Reconstruction was carried out through the NRecon software (version 1.6.10.2, Bruker microCT, Belgium). The reconstructed volumetric images were then imported in CTAn (CT Analyser version 1.14.4.1, Bruker MicroCT, Kontich, Belgium) and subjected to Otsu’s thresholding [32] to segment the pore volume. Porosity was then computed as the ratio of the pore volume to the total sample volume. For each dough recipe, three samples were scanned.

Compression tests were subsequently carried out by means of a universal testing machine (Stable Microsystems Texture Analyzer, Surrey, England) with a flat loading plate of 75 mm in diameter and a thickness of 10 mm. The measurements were performed with a load cell of 30 kg and a deformation rate of 0.1 mm/s. Stress–strain plots were obtained for each experiment, considering a logarithmic strain and a true stress rather than engineering values [33], and the Young modulus was then computed as the slope of the stress versus strain values (Table 1). For each recipe, nine samples were tested.

Recipe R1, whose composition is reported in Table 2, resulted in the best shape consistency after printing also marked by lowest total porosity. The Young modulus corresponding to the recipe R1 obtained from the compression was then used as input for models of mechanical deformation of cookie honeycomb structures together with a Poisson’s ratio of 0.45.

**Figure 2:**
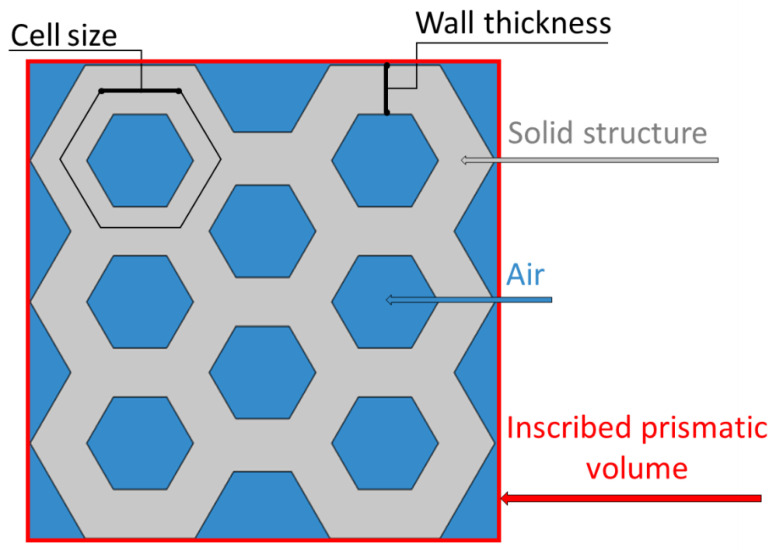
Cross-sectional view of the honeycomb structure with cell size of 7.5 mm and wall thickness of 2.6 mm. The structural features of the geometry are reported: the inscribed prismatic volume, air component, and solid structure are indicated in red, cyan, and grey, respectively. Porosity was computed as the air fraction relative to the inscribed prismatic volume.

For these models, geometries with different cell sizes and wall thickness were considered, as reported in Table 3. The porosity of the honeycomb structures was computed as the air fraction of the total structure volume which was defined as the volume of the structure inscribed prism [8] (Figure 2).

The honeycomb geometries reported in Table 3 were generated in AutoCAD (Autodesk, San Rafael, CA, USA) on a cubic domain with sides approximately equal to 20 mm and imported in COMSOL 4.3 (Comsol AB, Stockholm, Sweden). Here, the cookie geometries were discretized with tetrahedral elements. As result of a mesh sensitivity analysis, a maximum element size of 0.00025 m was used, corresponding to a maximum of 8,989,308 elements for the cookie with cell size 3.75 mm and wall thickness 2 mm (Table 3).

**Figure 3:**
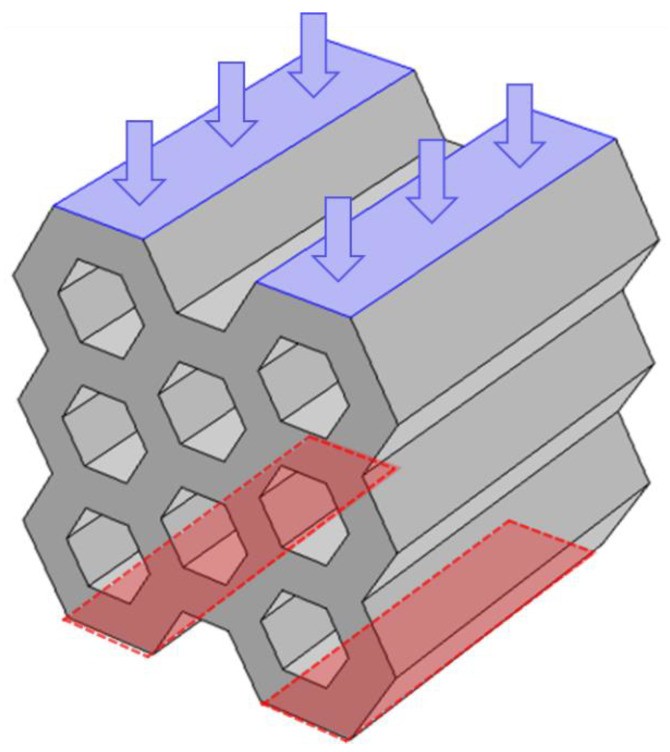
Representation of the simulation set-up. The upper boundary on which the vertical displacement was applied is represented in blue and the fixed bottom boundary in red.

Compression was then simulated by imposing a downwards vertical displacement of 3 mm to the upper boundary (Figure 3) of the models, while the bottom surface was modeled as a fixed boundary.

Simulations were performed in steady state and the displacement was applied in a linear perturbation step. The reaction force was evaluated on the upper boundary during displacement and used to compute the structure effective Young modulus as:

(1) E=σε=FAdLL=FLdL·A

where *E* (MPa) is the structure effective Young modulus, *σ* (MPa) the stress, and *ε* (-) the strain; *F* is the reaction force, *dL* (m) the deformation (i.e., the applied displacement), and *A* (m^2^) and *L* (m) the initial cross-section and length of the model, respectively. The effect of structure geometry on the effective Young modulus was then evaluated through the relative Young moduli of the samples:

(2) $$E^{*}=\frac{E}{E_{m}}$$

with *E_m_* the bulk material Young modulus, namely the Young modulus of the cookie dough obtained from recipe R1.

To validate the computational model, four honeycomb structures (Table 3) were 3D printed with dough recipe R1, and their mechanical properties were experimentally tested. Print files were generated and samples successfully 3D printed through EBP at room temperature and subsequently baked. The samples were printed on a custom TNO build extrusion setup using a piston to extrude dough in a syringe through a nozzle with diameter of 1 or 2 mm. During printing, the spreading of the bottom layers of the extruded dough could be observed. Details of the printing conditions used for the different samples, can be found in Table 4. For each geometry, 5 samples were 3D printed.

The 3D printed cookie samples were characterized in terms of structure, mechanical properties, and moisture content. The sample structure was investigated through X-ray µCT. For each selected geometry, three samples were scanned. The cookies were placed again in a µCT scanner Skyscan 1172 and scanned at 100 or 60 kV with a voxel size of 17.4 µm. After reconstruction, CT images were loaded in Avizo 9.3 (Visual Sciences Group, Bordeaux, France) for image processing.

**Figure 4:**
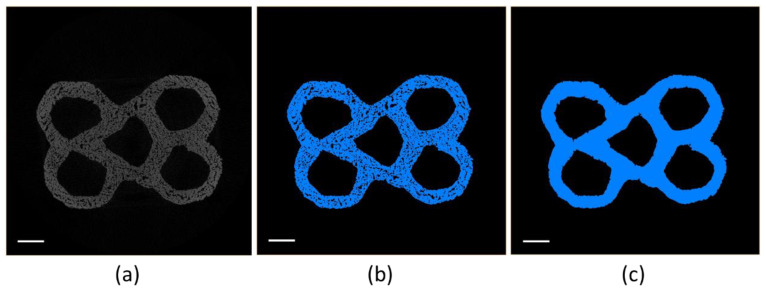
Representation of the image processing step for a honeycomb structure with cell size of 10 mm and wall thickness of 1.3 mm (H4): (**a**) cross-section of the original reconstructed volume, (**b**) automatic thresholding, (**c**) results of the “remove small holes” filter. The scale bars correspond to 5 mm.

A graphical representation of the image processing step is reported in Figure 4. The original reconstructed dataset (Figure 4a) was thresholded through automatic thresholding (Figure 4b) and subsequently filtered through the Avizo function “remove small holes”. As a result, the small pores belonging to the bulk material porosity (Figure 4c) were removed, as their presence was accounted for in the mechanical properties of the bulk material used in the FEM analysis (Table 1). Once the 3D printed structure was isolated, the volume of the structure inscribed prims was computed and the real 3D printing induced porosity was calculated.

After imaging, the samples were subjected to uniaxial in-plane compression tests to obtain their mechanical properties—i.e., Young’s modulus. Force-deformation measurements were carried out using a TA.XTPlus texture analyzer device (Stable Microsystems, Godalming, UK) with a load cell of 30 kg and maximal strain ε of 5% at a speed of 0.1 mm/s. The structure relative Young modulus was calculated through Equation (1) applied to the linear part of the stress–strain curves and Equation (2). The linear part of the stress–strain curve was estimated as a linear fit of the experimental data with a coefficient of regression higher than 0.95.

The moisture content of the cookies was quantified after the compression tests. Sample fragments were collected, weighed, and dried in an oven at a temperature of 103 °C for 16 h. Subsequently, the samples were weighed again, and the relative difference in weight before and after drying was regarded as the moisture content.

## 3. Results

### 3.1. Young’s Modulus Dependence on Geometry and Porosity

**Figure 5:**
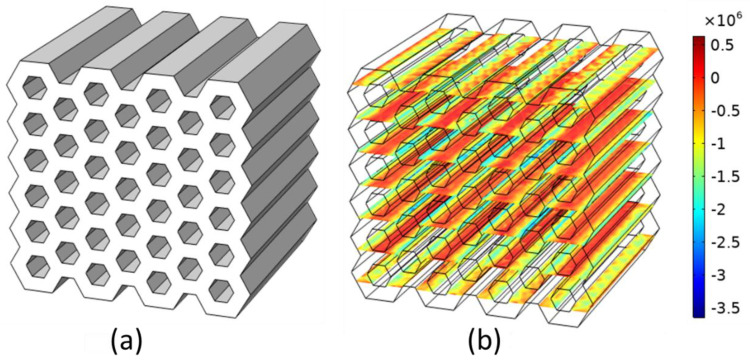
Representation of the compression simulations carried out to compute the sample Young’s modulus. (**a**) Model geometry for the honeycomb structure with wall thickness 1.3 mm and cell size 3.75 mm (H2) and (**b**) 3D plot of the stress *σ* (Pa) on the deformed geometry; an inhomogeneous stress distribution can be noticed.

Relative Young’s moduli were computed from FEM of the deformation of the honeycomb structures as illustrated in Figure 5.

**Figure 6:**
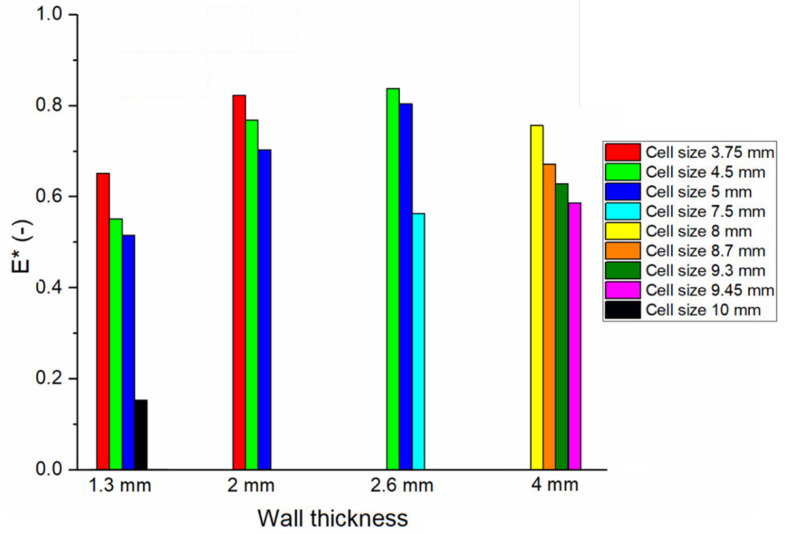
Relative Young’s modulus computed through FEM for designs with different wall thickness and cell size. Values increase with increasing wall thickness and decreasing cell size.

For the honeycomb structures, it was found that both cell size and wall thickness have an effect on the structure relative Young modulus, as shown in Figure 6. For designs with the same wall thickness, lower relative Young’s moduli were computed for larger honeycomb cell sizes. On the contrary, increasing wall thickness while maintaining the same cell size led to an increase in the sample relative Young modulus.

**Figure 7:**
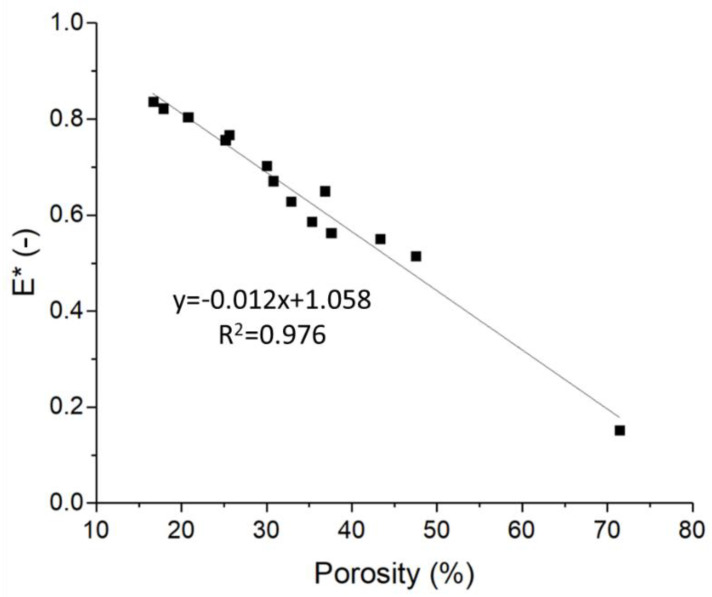
Values of the relative Young’s modulus with respect to the sample porosity. An inverse linear dependence on porosity can be observed, regardless of the sample wall thickness and cell size.

Moreover, a dependence of the relative Young modulus on the sample porosity was observed, regardless of wall thickness and cell size, as depicted in Figure 7. The relative Young modulus was found to decrease linearly (linear fit equation reported in Figure 7) with increasing sample porosity, with a regression coefficient of 0.976.

### 3.2. Characterization of 3D Printed Cookies

The 3D printed cookies visually deviated from the original designs, as a result of the printing process and in particular as a consequence of deformation during baking. To quantify the structure of the actual 3D printed products, cookies were characterized by X-ray µCT and the results can be found in Table 5. For all the samples, a lower porosity was measured with respect to the design porosity with a maximum relative difference of 62.8% measured for sample H2.

**Figure 8:**
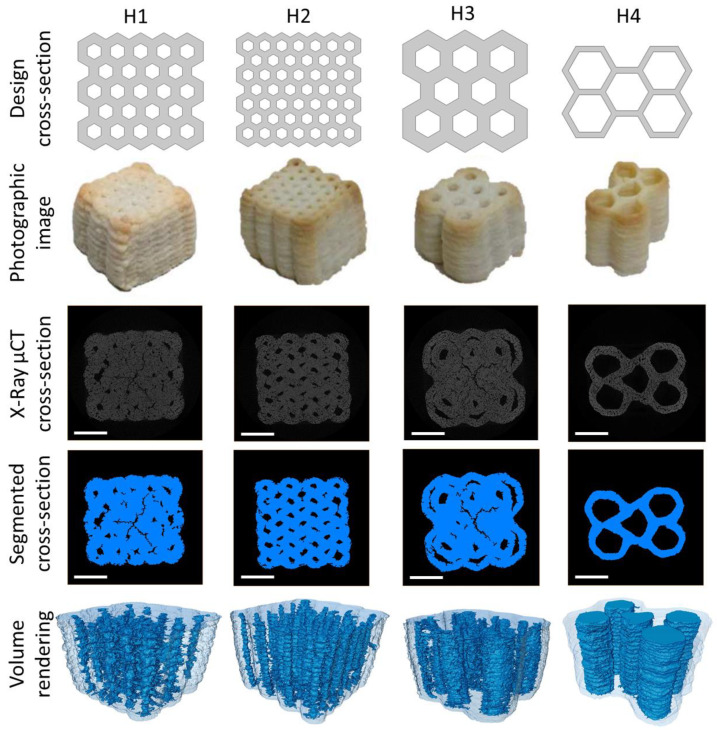
Cross-section design (**row 1**), photographic image (**row 2**), X-ray µCT image slice (**row 3**), segmented X-ray µCT image slice (**row 4**), and volume rendering (**row 5**) of the four experimentally tested honeycomb structures. The scale bar of µCT images corresponds to 10 mm.

From the µCT images (Figure 8), the presence of cracks that occurred in some samples during the baking process can be noticed. These cracks show an anisotropic orientation and might influence the structure stability and Young modulus. Moreover, detachment of the wall following baking can be observed for sample H3 as a consequence of the use of a too small nozzle, requiring construction of this design based on double walls. An increased wall thickness with respect to the original design can be observed in the µCT images, as a consequence of the spreading of cookie dough during printing and of the baking process. This geometry modification clearly led to a pore size reduction and ultimately to a lower measured porosity. From the µCT images, it can also be noticed how the 3D printed samples had a more rounded geometry than the original honeycomb structure design, with a reduction in the sharpness of the geometry edges.

### 3.3. Validation of FEM Predicted Young’s Modulus

**Figure 9:**
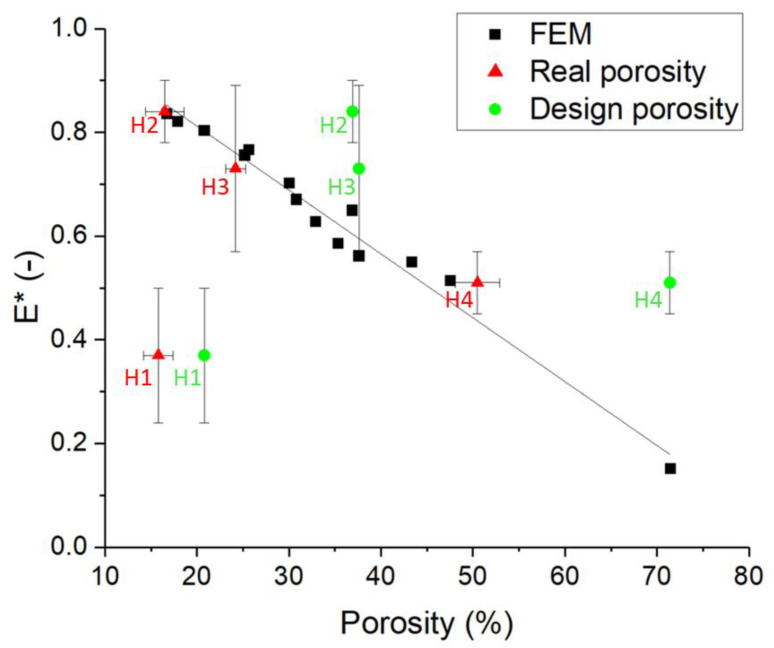
Comparison of the relative Young’s modulus of the 3D printed cookie honeycomb structures obtained from experimental tests and from FEM. Experimental results are reported as function of the design porosity (green circles) and of the measured porosity (red triangles).

Relative Young’s modulus values obtained from the experimental characterization performed on the 3D printed samples are reported in Table 5. These values were compared to the those predicted through FEM simulations. Figure 9 reports predicted and measured values together; measured Young’s moduli are plotted as function of both the design and measured porosity. The analysis shows that a much better agreement was generally found between FEM predicted results and experimental values when the real porosity measured through X-ray µCT was taken into account. Considering the design sample porosity mostly resulted in a strong overestimation of the sample Young moduli. It can also be noticed that the measured Young modulus for sample H1 (cell size of 5 mm and wall thickness of 2.6 mm) remains significantly lower than the FEM predicted value, both considering the predicted and the real sample porosity. This deviation is expected to be a consequence of the high moisture content reported for sample H1, as further discussed in the following section.

## 4. Discussion

From the FEM results (Figure 6) it was observed that the Young modulus of the 3D printed cookie structures was strongly dependent on both the wall thickness and honeycomb cell size. Thicker walls and smaller cells granted the modelled structures higher stiffness and stability and thus a higher Young’s modulus. On the contrary, geometries with thin walls and large cells were characterized by a lower Young’s modulus. These results clearly indicate that both wall thickness and cell size can be used as design parameters to engineer and control the texture of 3D printed cookies as represented by their Young modulus. Nevertheless, through X-ray µCT scans, it was observed that the honeycomb’s original design was subject to important modifications during the 3D printing and baking processes (Figure 8). In particular, a thickening of the walls was observed, most probably due to the dough spread during printing and to the dough expansion during baking. These changes in the structure design led to smaller cells and, therefore, to a reduction in the cookie porosity in all designs. From the µCT scan results, the importance of properly selecting the printing nozzle was also evident. H3 samples were 3D printed with a 1 mm wide nozzle, requiring two passages to create the thick cell walls, which resulted in wall separation during the baking process. H1 samples, whose wall thickness was the same as for H3 samples, were 3D printed with a 2 mm diameter nozzle and did not experience wall separation during baking. This result suggests that the nozzle should be adapted as a function of the wall thickness, avoiding to print thick walls with narrow nozzles.

Interestingly, the FEM results also showed an inverse linear dependence of the structure’s Young’s moduli to the design porosity, regardless of wall thickness and cell size, with low porosity samples being characterized by high Young’s moduli. This linear relationship was characterized (Figure 7) and could be used as starting point to produce 3D printed cookies with desired Young’s modulus by controlling the cookie porosity.

When comparing the FEM results to the experimentally obtained Young moduli of the 3D printed samples, large deviations from the modelled values can be observed when the sample design porosity is considered. In this case, the experimental Young moduli were mostly higher than the modelled ones (Figure 9). This is probably due to porosity differences between the structure designs and the final 3D printed samples. Indeed, when the actual porosity computed from X-ray µCT scans was considered, a significantly better match between modelled and experimental Young’s modulus values could be observed (Figure 9). Nevertheless, H1 samples exhibited a much lower Young’s modulus than expected. This could be a consequence of the cracks arising in the samples during baking (Figure 8). While these cracks did not contribute significantly to the sample porosity, they might have influenced the structure stability, especially when oriented perpendicularly to the compression plane, leading to a decrease in Young’s modulus [34]. Computational modelling of mechanical compression performed on the real cookie geometry obtained from CT scans (Figure 8), however, showed that the cracks did not greatly affect the structure of the Young modulus (results not shown). Another explanation for the low Young modulus can be found in the high moisture content (9.03 ± 1.19%) measured for these samples after baking, as it is known that, for cookies, the Young modulus rapidly declines when the moisture content is higher than 6% [34]. The final moisture content after baking was also found to be structure dependent, as all the other samples showed a moisture content lower than 4%, with sample H4 exhibiting the lowest moisture content (Table 5). The moisture content is linked to the cookie sample porosity as changing the internal geometry affects the mass transport during baking and thus the baking kinetics itself [35]. As a result, low porosity cookies show a higher moisture content after baking [35]. This effect significantly complicates model-based design of 3D printed cookies as porosity not only determines the cookie structure Young modulus, but also the moisture content, which in turn influences the Young modulus.

Future work should, therefore, focus on understanding how the moisture content effects the Young modulus of the cookie dough. Moreover, studying the dependency of the final moisture content on the cookie porosity is essential to determine a priori the Young modulus of the bulk material to be used for CAE-based design. Finally, the Young modulus of the 3D printed cookie was found to be a function of the real sample porosity. For this reason, predicting geometry modifications of 3D printed cookies during printing and baking, for example through modelling [36,37,38], is crucial to successfully estimate the resulting cookie properties. Successful modelling of the baking process would thus be valuable to predict both the final cookie geometry and water content and, in turn, effectively predict the cookies’ Young modulus. By including modelling of hygro-mechanical stresses arising during baking, it would also be possible to predict the formation of cracks [39,40]. Nevertheless, the amount of unwanted cracks in the 3D printed cookies should be minimized through optimization of the dough composition and the baking processes to increase the reliability of the FEM predictions.

## 5. Conclusions

In this study, FEM was used to investigate the mechanical properties—i.e., Young modulus—of cookies with honeycomb structure obtained through EBP. The model showed that wall thickness and cell size can be used as design parameters to produce 3D printed cookies with different Young’s moduli through a CAE approach. Moreover, an inverse linear relationship was found and quantified between the sample Young modulus and porosity. Experimental results showed a good agreement with FEM results when the real cookie porosity was considered. Since structure modifications cannot be completely avoided, future research should focus on predicting the final cookie geometry after printing and baking based on a model of these processes. In this way, cookie mechanical properties could be reliably designed.

## Figures and Tables

**Table 1:** Summary of the properties of the different cookie dough recipes used for the compression test.

Dough Recipe	Baking Powder Concentration	Total Porosity [%]	Young’s Modulus [MPa]
R1	None	33.03 ± 0.69	8.29 ± 1.72
R2	Medium	56.61 ± 1.14	7.58 ± 0.84
R3	High	59.43 ± 3.10	5.56 ± 1.12

**Table 2:** Composition of the dough recipe R1.

Component	Quantity [%]
Flour	48.43
Sugar	9.69
Polydextrose	9.69
Margarine	24.21
Egg protein powder	2.42
Water	4.84
Emulsifier Panodan	0.48
Vanilla sugar	0.24

**Table 3:** Summary of the geometries studied through FEM with their respective porosity values (in the table cells). Geometries marked with * were 3D printed and experimentally tested.

	Cell Size [mm]									
**Wall thickness [mm]**		**3.75**	**4.5**	**5**	**7.5**	**8**	**8.7**	**9.3**	**9.45**	**10**
	1.3	36.9% *	43.3%	47.5%						71.4% *
	2	16.7%	25.6%	30.0%						
	2.6		17.9%	20.8% *	37.6% *					
	4					25.2%	32.9%	35.3%	30.8%	

**Table 4:** Printing parameters used to 3D print the experimental samples.

Sample	Cell Size [mm]	Wall Thickness [mm]	Nozzle Diameter [mm]	Printing Speed [mm/min]	Layer Height [mm]	Extrusion Amount [mm^3^/Printed mm]
H1	5	2.6	2	400	1	2.14
H2	3.75	1.3	1	300	0.5	0.8
H3	7.5	2.6	1	300	0.5	0.8
H4	10	1.3	1	200	0.5	0.8

**Table 5:** Summary of samples characterized by X-ray µCT. For all samples, a reduction in porosity can be observed.

Sample	Cell Size [mm]	Wall Thickness [mm]	Design Porosity	Real Porosity	Moisture Content	Relative Young’s Modulus [MPa]
H1	5	2.6	20.8%	15.8 ± 1.6%	9.03 ± 1.19%	0.37 ± 0.13
H2	3.75	1.3	36.9%	16.5 ± 2.1%	3.6 ± 0.085%	0.84 ± 0.06
H3	7.5	2.6	37.6%	24.2 ± 1.1%	3.53 ± 0.13%	0.73 ± 0.16
H4	10	1.3	71.4%	50.5 ± 2.4%	3.41 ± 0.25%	0.51 ± 0.06
